# Pathways to ovarian cancer diagnosis: a qualitative study

**DOI:** 10.1186/s12905-022-02016-1

**Published:** 2022-11-04

**Authors:** Katherine A. Lawson-Michod, Melissa H. Watt, Laurie Grieshober, Sarah E. Green, Lea Karabegovic, Samantha Derzon, Makelle Owens, Rachel D. McCarty, Jennifer A. Doherty, Mollie E. Barnard

**Affiliations:** 1 Huntsman Cancer Institute, University of Utah, 2000 Cir of Hope Dr, Salt Lake City, UT 84112 USA; 2 University of Utah Intermountain Healthcare Department of Population Health Sciences, School of Medicine, University of Utah, 295 Chipeta Way, Salt Lake City, UT 84108 USA; 3 Department of Internal Medicine, School of Medicine, University of Utah, 30 N 1900 E, Salt Lake City, UT 84132 USA; 4 Danbury Hospital Department of Surgery, Danbury Hospital, 24 Hospital Ave, Danbury, CT 06810 USA; 5 Utah Valley Hospital, Utah Valley Family Medicine Residency, Intermountain Healthcare, 475 W 940 N, Provo, UT 84604 USA; 6 San Antonio Military Medical Center Internal Medicine Residency, Brooke Army Medical Center, 3551 Roger Brooke Dr, San Antonio, TX 78234 USA; 7 Slone Epidemiology Center, Boston University Chobanian & Avedisian School of Medicine, 72 East Concord St., Boston, MA 02118 USA

**Keywords:** Ovarian cancer, Qualitative research, Access to healthcare, Patient education, Delayed diagnosis

## Abstract

**Background:**

Ovarian cancer is often diagnosed at a late stage, when survival is poor. Qualitative narratives of patients’ pathways to ovarian cancer diagnoses may identify opportunities for earlier cancer detection and, consequently, earlier stage at diagnosis.

**Methods:**

We conducted semi-structured interviews of ovarian cancer patients and survivors (*n* = 14) and healthcare providers (*n* = 11) between 10/2019 and 10/2021. Interviews focused on the time leading up to an ovarian cancer diagnosis. Thematic analysis was conducted by two independent reviewers using a two-phase deductive and inductive coding approach. Deductive coding used a priori time intervals from the validated Model of Pathways to Treatment (MPT), including self-appraisal and management of symptoms, medical help-seeking, diagnosis, and pre-treatment. Inductive coding identified common themes within each stage of the MPT across patient and provider interviews.

**Results:**

The median age at ovarian cancer diagnosis was 61.5 years (range, 29–78 years), and the majority of participants (11/14) were diagnosed with advanced-stage disease. The median time from first symptom to initiation of treatment was 2.8 months (range, 19 days to 4.7 years). The appraisal and help-seeking intervals contributed the greatest delays in time-to-diagnosis for ovarian cancer. Nonspecific symptoms, perceptions of health and aging, avoidant coping strategies, symptom embarrassment, and concerns about potential judgment from providers prolonged the appraisal and help-seeking intervals. Patients and providers also emphasized access to care, including financial access, as critical to a timely diagnosis.

**Conclusion:**

Interventions are urgently needed to reduce ovarian cancer morbidity and mortality. Population-level screening remains unlikely to improve ovarian cancer survival, but findings from our study suggest that developing interventions to improve self-appraisal of symptoms and reduce barriers to help-seeking could reduce time-to-diagnosis for ovarian cancer. Affordability of care and insurance may be particularly important for ovarian cancer patients diagnosed in the United States.

**Supplementary Information:**

The online version contains supplementary material available at 10.1186/s12905-022-02016-1.

## Background

Nearly 60% of epithelial ovarian cancers are diagnosed at a late stage, at which time five-year survival is only 29% [5]. In contrast, for the 15% of ovarian cancers diagnosed at a localized stage, five-year survival is 92% [5]. Stage shifts and subsequent improvements in patient survival have been accomplished through population-level screening for common cancer types (e.g., breast, colon) [3, 4, 11, 30, 34, 35, 51]. However, for a rare cancer like ovarian cancer, a population-level screening test would need to have near perfect sensitivity and specificity for the benefits of the test to outweigh the risks. To date, no population-level screening tests, symptom indices, or other early detection tools have successfully improved ovarian cancer survival [6, 9, 13, 14, 20, 26, 31]. Novel interventions are needed to reduce the time-to-diagnosis for ovarian cancer and improve survival; however, the pathway to an ovarian cancer diagnosis has not been fully characterized and opportunities for reducing time-to-diagnosis remain understudied.

Quantitative studies have begun to characterize the time leading up to cancer diagnoses [1, 8, 15, 19, 21, 23, 27, 47]. For ovarian cancer, time-to-diagnosis is observed to differ by disease histotype [27], and is associated with race, ethnicity, US geographic region, presenting symptom, and specialist type initially consulted [19]. While the majority of ovarian cancer patients first present to their primary care physician [19, 27], delays in diagnosis are common irrespective of the provider specialty initially consulted [1, 19, 23, 47]. For example, one Medicare-based study evaluating factors influencing time-to-diagnosis for ovarian cancer estimated that 30% of their study population consulted more than 4 specialists prior to diagnosis [19], and case-only studies from Denmark and the UK described an increase in medical encounters during the year leading up to an ovarian cancer diagnosis [1, 23, 47]. Population-based studies out of Denmark also reported changes in medical encounters in the months leading up to any cancer diagnosis, including an increase in the number of primary care visits and a greater frequency of switching primary care providers [8, 15, 21]. Among the 23 cancer types investigated, changes in primary care providers were most common among ovarian cancer patients [15]. These studies indicate that many cancer patients, particularly ovarian cancer patients, struggle to obtain timely and accurate diagnoses.

While record-based studies have identified a number of healthcare system factors that influence time-to-diagnosis [19, 27], their reliance on medical records excludes the time prior to when an individual enters the healthcare system. Qualitative studies have the potential to characterize the time prior to entering the healthcare system, thereby improving our understanding of factors impacting patients’ evaluation of symptoms, and how, where, and when patients decide to seek care [12, 28, 49]. For example, studies from the UK and Denmark reported that patient normalization of symptoms, scheduling conflicts, and limited availability of general practitioners delay time-to-diagnosis [12, 28]. It is important to understand how these early barriers and facilitators of help-seeking behavior translate to a multi-payer system, like the US healthcare system.

**Fig. 1:**
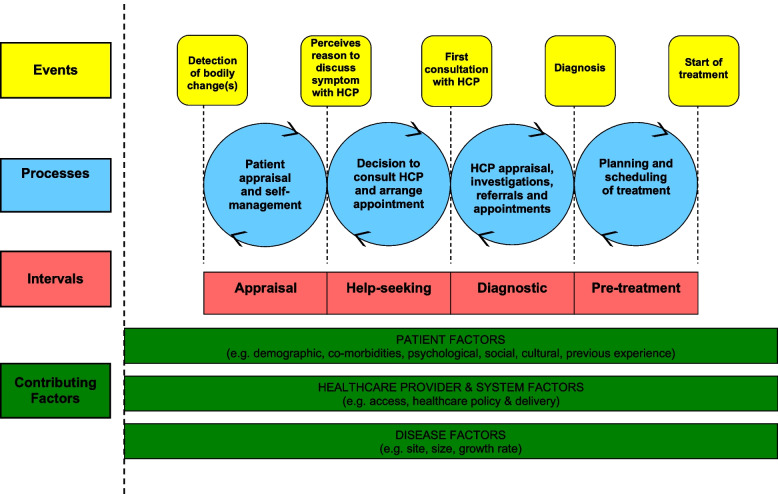
Model of pathways to treatment (MPT): A validated framework for evaluating pathways to diagnosis. Reproduced with permission from the publisher and authors of Scott et al.*,* 2012 [32]

Further characterizing the complete series of events leading up to the initial treatment of ovarian cancer in a multi-payer healthcare system could inform effective interventions for reducing time-to-diagnosis and treatment, which could contribute to improved quality of life and survival duration. The Model of Pathways to Treatment (MPT) is a validated framework that has been used to analyze patient-reported pathways to diagnosis and initial receipt of treatment (including for cancer [2, 7, 18, 37, 50]), to identify barriers and facilitators to timely diagnosis and treatment, and to characterize patient perceptions of pathways to diagnosis and treatment [2, 18, 43]. The MPT includes self-appraisal, help-seeking, diagnostic, and pre-treatment intervals (Fig. 1). While prior qualitative studies have focused on the perspectives of ovarian cancer patients who were diagnosed and cared for in countries with single-payer or universal healthcare systems, we leveraged the MPT framework to synthesize both patient and provider perspectives on factors that influence time to diagnosis and treatment in a US-based, multi-payer healthcare setting.

## Materials and methods

### Design

We conducted a qualitative study of patient and provider perspectives on pathways to ovarian cancer diagnosis using semi-structured interviews and two-phase analysis. This study was conducted at the Huntsman Cancer Institute, a National Cancer Institute-designated Comprehensive Cancer Center serving Utah, Idaho, Montana, Nevada, and Wyoming [53]. Reporting of the qualitative methods and findings follow the Consolidated Criteria for Reporting Qualitative Research (COREQ) guidelines [48].

### Participants

Ovarian cancer patients diagnosed or treated at the University of Utah from 2/5/2018 to 8/6/2020 who opted to participate in the “Huntsman Cancer Institute Total Cancer Care Study (TCC),” [17] and healthcare providers with University of Utah affiliation at the time of interview, were eligible for the study. Non-probability sampling methods, including purposive and convenience sampling, were used to identify eligible patients and providers.

Eligible patients were identified by chart review of TCC-consented patients within the University of Utah’s electronic health record system (EHR). All patients were 18 years of age or older, and no patients were excluded based on gender, race, ethnicity, stage at diagnosis, or ovarian tumor type. We sought to include patients from multiple locations across the Huntsman Cancer Institute catchment area, including patients not living in immediate proximity to the University of Utah.

For providers we sought to include primary care doctors and specialists who may interact with ovarian cancer patients during the patient path to diagnosis. We first identified providers through chart review of participating patients. This screening method returned limited results; thus, additional providers were identified through the University of Utah “Find a Doctor” tool. In addition to primary care and oncology, we targeted providers from common specialties, including cardiology, emergency medicine, and urgent care; and specialties with symptomology similar to ovarian cancer, including gastroenterology, pulmonology, and neurology.

Eligible patients and providers received an email introducing the research study and a subsequent telephone call inviting them to participate. Of 32 eligible patients contacted, 14 chose to participate (44%), 13 did not respond (40%), and 5 declined (16%). Of 174 healthcare providers contacted, 11 agreed to participate (6%), 156 (90%) did not respond, and 7 declined (4%).

### Data collection

Trained study staff conducted telephone-based interviews of ovarian cancer patients and survivors (KLM, LK) and healthcare providers (KLM, SEG, MO) from 10/18/2019 to 10/10/2021. All interviewers were female doctoral or medical students. At the start of each interview, the interviewer restated the research objectives and participants were given the opportunity to ask questions. The median duration for patient interviews was 25 minutes (range: 12–38 minutes) and for provider interviews was 23 minutes (range: 16–52 minutes). Participants were not re-contacted following the interview.

Using a semi-structured interview guide (Supplementary Information 1), patients were asked to tell their cancer story with particular emphasis on their experiences leading up to their diagnosis. Follow-up questions were intended to gather information on symptom descriptions and timing, encounters with medical and alternative care providers, medication use, diagnostic tests, and quality of care. Patients were specifically asked about the year prior to their diagnosis, but experiences outside of that range were also discussed.

Semi-structured provider interviews (Supplementary Information 2) focused on ovarian cancer symptoms and patient pathways to diagnosis. Providers who had ever participated in the referral pathway for an ovarian cancer diagnosis were asked about their personal experiences, while other providers were asked about their perceptions of patient pathways to an ovarian cancer diagnosis. Providers were additionally asked about their perceptions of ovarian cancer patients’ barriers to receiving their diagnosis.

Trustworthiness of the data was assured in the data collection process by piloting the interview questions, and conducting in-depth interviewer training which included 2–3 practice interviews and peer debriefing following interviews. Semi-structured interview questions were written by two postdoctoral fellows (MEB and LG) and piloted among a group of cancer-free individuals. All interviews were audio-recorded and transcribed following the interview. Transcription was completed using the Microsoft 365 automated transcribe tool with subsequent manual review by study staff.

Patients’ demographic and tumor characteristics and the dates of patients’ first visits to Huntsman Cancer Institute were abstracted from the University of Utah’s electronic health record. The duration of each MPT interval (Fig. 1) was estimated from patient interviews in concordance with the Aarhus guidelines [52]. From the patient interviews, we also abstracted patient-reported symptoms and the specialties of providers they consulted prior to diagnosis.

### Data analysis

**Fig. 2:**
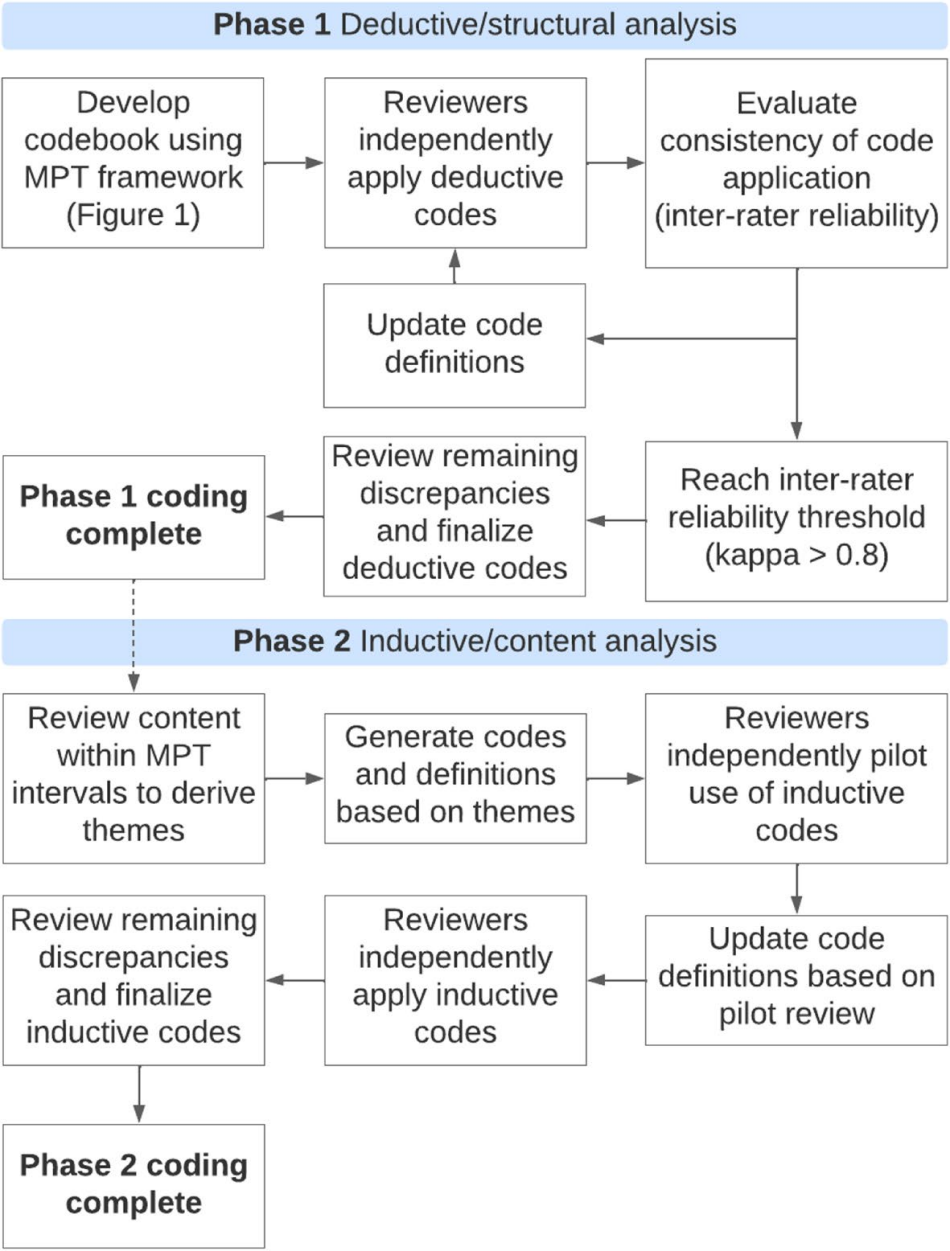
Approach to qualitative thematic analysis using a two-phase deductive and inductive approach

We performed a qualitative thematic analysis using a two-phase deductive and inductive coding approach (Fig. 2). Deductive coding was completed by two independent reviewers (KLM and MEB) using a priori codes from the validated MPT framework. The MPT delineates a patient’s pathway through self-appraisal and self-management of symptoms, help-seeking, the diagnostic process, and planning and scheduling of treatment (Fig. 1). Deductive coding was an iterative process, and coders met frequently to discuss discrepancies and refine MPT interval definitions to improve consistency and relevance. We evaluated inter-rater reliability after each codebook update, and persisted with reapplication of deductive codes until a threshold kappa of 0.8 was reached (final kappa = 0.825). As a final step in deductive coding, the coders discussed all remaining discrepancies until they reached a consensus. Inductive coding was completed by the same independent reviewers who re-reviewed all transcripts to derive themes within each of the MPT intervals. Following the preliminary identification of themes, the reviewers met to refine the codebook. Codes for the inductive themes were created as child codes under the deductive codes (Table 1). During inductive coding, no themes were added to the codebook with the analysis of the final four transcripts indicating data saturation [42]. The reviewers independently applied inductive codes and discussed any discrepancies until a consensus was reached. Trustworthiness of the data was assured in the data analysis process by having two independent analysts code the data and discuss discrepancies until a consensus was reached. All deductive and inductive coding was completed in Dedoose (version 9.0.46).

**Table 1:** Codebook that was used for the qualitative thematic analysis

Deductive Code	Inductive Theme	Sub-Themes
**Appraisal**	Access to care	Established doctor-patient relationships; continuity of care
	Avoidant coping strategy	Fear of a serious medical diagnosis
	Perception of underlying health	
	Concerns about the patient-provider relationship	Fear of judgment from provider
	Nonspecific symptoms	
	Management and normalization of symptoms	Menopause; aging; benign condition; existing medical condition
**Help-Seeking**	Access to care	Bringing up symptoms because of routine healthcare visits; continuity of care; insurance; cost of care; provider availability; patient work or school schedule
	Avoidant coping strategy	Fear of a serious medical diagnosis or of medical tests
	Persistence in seeking care	
	Perception of underlying health	
	Concerns about the patient-provider relationship	Dislike of doctors; concern of judgement from providers; feeling comfortable with a provider; embarrassment surrounding symptoms
	Social support	
	Worsening, extreme, or abnormal symptoms	
**Diagnostic**	Access to care	Continuity of care; quality of care (referrals and workup); accessibility (transportation; distance to healthcare facility); availability (provider availability; ability to schedule around other responsibilities, e.g., work and school); affordability of care
	Provider perception of patient risk	
	Features of rapid diagnoses	
	Features of complex diagnoses	
**Pre-Treatment**	Access to care	Quality of care (method of receipt of diagnosis, established referral pathways between referring physician and gynecologic oncology); accessibility and availability of care (rapid referrals, distance to cancer center, transportation and lodging); affordability; education; language
	Social support	

### Ethical considerations

This study was reviewed and deemed “exempt” by the University of Utah Institutional Review Board (Reference Number 00123466). All patient participants had previously provided written informed consent agreeing to participate in TCC. Additionally, all patient and provider participants received a study-specific consent letter by email, and gave verbal consent to participate in this study at the time of the interview. As noted above, the research objectives were restated before starting the interview. Telephone interviews took place with the interviewer in a private room (office or at home during COVID-19) and the interviewee in a place they felt comfortable answering questions. HIPAA compliant data storage spaces and data analysis tools were used.

## Results

Of the 14 participating ovarian cancer patients and survivors, the median age at ovarian cancer diagnosis was 61.5 years (range: 29–78 years), and the majority of participants (11/14) were diagnosed with advanced stage disease [International Federation of Obstetrics and Gynecology (FIGO) Stage III or IV]. The median time from first symptoms to diagnosis was 2.8 months (range: 19 days to 4.7 years). Interviews of patients occurred a median of 15 months (range: 7 months to 3.4 years) following the initial ovarian cancer diagnosis. The 11 participating providers represented the following specialties: cardiothoracic surgery (*n* = 2), primary care (*n* = 2), emergency medicine (*n* = 2), gastroenterology (*n* = 2), medical oncology (*n* = 1), pulmonology (*n* = 1), and neurology (*n* = 1).

The appraisal duration, the time from a patient’s first detection of a symptom to their decision to consult a healthcare provider, and the help-seeking duration, the time from the decision to consult a healthcare provider to the patient’s first visit with a healthcare provider, were not consistently distinguishable in patient reports, so these durations were combined (Table 2). The median combined appraisal and help-seeking interval was 2.5 months (range: 7 days to 4.5 years), while the median time from first healthcare consult to ovarian cancer diagnosis (diagnostic) was 18 days (range: 0 days to 3.75 years), and the median time from ovarian cancer diagnosis to start of treatment was 8 days (range: 1 day to 1 month).

**Table 2:** Ovarian cancer patient characteristics, key features of patient paths to diagnosis, and duration of MPT intervals

ID	Age^**a**^	Stage^**a**^	Histology	Routine healthcare^**b**^	Referral pathway^**c**^	Symptoms	Providers consulted	Appraisal and help-seeking duration (months)	Diagnostic interval duration (months)	Pre-treatment interval duration (months)	Total MPT interval duration (months)	Time from diagnosis to interview (years)
5	55	IV	SC NOS	Yes	Secondary	FS: urinary frequency RFH: urinary frequency, incontinence, bloating, back pain, palpable mass	1st gastroenterologist, naturopath, 2nd gastroenterologist (×2)	12.17	45	0.13	57.30	2.66
4	64	IV	SC NOS	No	Secondary	FS: abdominal pain, bloating and flatulence, RFH: lump in armpit	PCP, general surgeon, radiologist	12.17	0.63	0.33	13.13	2.82
1	66	IV	HGSC	Yes	Direct	RFH: rapid weight gain	PCP	0.23	NE	0.40	0.63	1.42
12	78	IV	SC NOS	No	Secondary	FS: weight gain RFH: abdominal pain	ED physician, outpatient physician NOS	12	0.33	0.20	12.53	1.33
11	68	IV	HGSC	Yes	Secondary	RFH: difficulty breathing	PCP, ED physician	3	0.23	1.00	4.23	0.71
9	70	IV	C NOS	Yes	Secondary	FS/RFH: neck mass	PCP, ENT	1.37	0.6	0.03	2.00	0.84
10	69	IV	HGSC	Yes	Secondary	RFH: difficulty breathing	Urgent care provider, ED physician, PCP	NA	0.25	0.40	0.65	0.62
13	29	III	Dys	Yes	Direct	FS: tongue sores (“immune reaction”) RFH: abnormal periods, weight gain	PCP	29.43	12	0.20	41.63	0.80
14	72	III	HGSC	Yes	Secondary	RFH: bladder discomfort	Gynecologist, PCP, initial gynecologist	2	0.97	0.13	3.10	1.12
8	48	III	Dys	Yes	Secondary	FS/RFH: cough	Urgent care provider, PCP, pulmonologist	0.5	1.9	0.03	2.43	1.30
15	59	III	HGSC	Yes	Direct	RFH: abdominal pain	PCP	NA	0.75	0.40	1.15	1.13
2	53	II	SC NOS	Yes	Direct	FS: urinary incontinence, back pain RFH: vaginal bleeding	Midwife	0.33	0.17	0.43	0.93	2.21
7	46	I	SC NOS	Yes	ED Direct	FS: urinary incontinence, back pain RFH: back pain, nausea	Massage therapist, ED physician	54	0	0.57	54.57	0.79
6	57	I	EA NOS	No	ED Direct	FS: abdominal pain RFH: worsening of abdominal pain, and dry heaving	ED physician	1.1	0.1	0.03	1.23	3.36

*Abbreviations*: *Unk* Unknown, *HGSC* high grade serous carcinoma, *SC* serous carcinoma, *EA* endometrioid adenocarcinoma, *Dys* dysgerminoma, *C* carcinoma, *NOS* not otherwise specified, *RFH* reason for help-seeking, *FS* first symptom, *PCP* primary care provider, *ED* emergency department, *ENT* ear nose and throat, *NE* could not estimate based on transcripts, *NA* not applicable, *Dx* diagnosis. ^a^Age and stage defined at diagnosis. ^b^Routine healthcare defined by seeing a health care provider for routine care within 12 months of diagnosis. ^c^Referral pathways were defined as follows: “direct” patients were seen by an outpatient provider and directly referred by the same provider to gynecologic oncology; “secondary” patients consulted with ≥2 providers prior to receiving a referral to gynecologic oncology; “ED direct” patients first sought help in the emergency department and were directly referred from the ED to gynecologic oncology. Durations of the MPT intervals (Columns 9–13) were estimated from patient narratives. The aggregate appraisal and help-seeking interval was estimated from a patient’s first detection of a symptom to consult with a healthcare provider. The diagnostic interval was defined from first consult with a healthcare provider to receipt of ovarian cancer diagnosis. The pre-treatment interval was defined from the time of ovarian cancer diagnosis to the start of treatment

### Appraisal

Common themes identified across patients and providers during the appraisal interval included nonspecific symptoms, management and normalization of symptoms, perception of underlying health, an avoidant coping strategy, access to care, and concerns about the patient-provider relationship (Tables 1 and 3).

**Table 3:** Additional representative quotes from patients and providers identified during two-phase qualitative analysis

MPT Interval	Inductive Theme	Quote
Appraisal	Nonspecific symptoms	*“Mild abdominal pain, changes in bowel movements, nausea, change in the size of the abdomen without frank distention, sometimes pulmonary symptoms if someone has malignant ascites, urinary symptoms, early satiety ... a broad range of often vague and nonspecific symptoms.” (Provider 1, Internist)*
Appraisal	Nonspecific symptoms	*“Ovarian cancer is obviously one of those things where symptoms are not very reliable, as is true for most cancers.” (Provider 3, Gastroenterologist)*
Appraisal	Nonspecific symptoms; Management and normalization of symptoms; Perception of underlying health	*“... and the bloating and stuff ... I really thought two things were going on. I thought I was having some menopausal issues even though I had had a hysterectomy ... I’d been doing hormone replacement therapy with estradiol and low-dose testosterone, but I wasn’t doing progesterone, so I thought maybe these were just symptoms of menopause because I’m, you know, older.” (Patient 5, Age 55, Stage IV)*
Appraisal	Nonspecific symptoms; Management and normalization of symptoms	*“I was mostly just tired and achy, and you could be tired and achy for a billion different reasons.” (Patient 13, Age 29, Stage III)*
Appraisal	Management and normalization of symptoms; Perception of underlying health	*“I never would have thought of ovarian cancer. I never would have thought of cancer. I just thought for sure I was never getting cancer ‘cause I was too healthy. My lifestyle was everything about not cancer. I did water fasting. I ate a clean diet--it was ketogenic half the time.” (Patient 5, Age 55, Stage IV)*
Appraisal	Management and normalization of symptoms	*“I would eat yogurt to calm my intestines.” (Patient 4, Age 64, Stage IV)*
Appraisal	Perception of underlying health	*“I had just gone in for my yearly exam[s] … pap smear, the dermatologist, my eye exam. I was so on the ball.” (Patient 8, Age 48, Stage III)*
Appraisal; Help-seeking	Avoidant coping strategy; Social support	*“Sometimes when you probe the patients a little further, especially the ones that don’t want to come in ... they’ll say, ‘I knew something was wrong and I didn’t want to know’ or ‘my family member actually made me come in’.” (Provider 2, Oncologist)*
Appraisal; Help-seeking	Avoidant coping strategy	*“I remember looking up stomach pain, and it did say that’s one thing you should not discount…but I thought well, my biggest fear was tests. I didn’t want to have to go through tests, ‘cause they wouldn’t be able to diagnose my problem.” (Patient 6, Age 57, Stage I)*
Appraisal; Help-seeking	Access to care	*“I’m very cheap and that’s one of the reasons I didn’t go to the doctor’s, ‘cause I’m like, I don’t want to pay for this, you know, all these tests ‘cause I know tests are expensive.” (Patient 6, Age 57, Stage I)*
Help-seeking	Worsening, extreme, or abnormal symptoms; Persistence in seeking care	*“I texted [my PCP, a nurse practitioner,] and said that [the symptoms were] getting worse.” (Patient 15, Age 59, Stage III)*
Help-seeking	Access to care	*“I was due to see my midwife for my annual exam anyway, so I called her.” (Patient 2, Age 53, Stage II)*
Help-seeking	Access to care	*“Their medical literacy and I’m sure their education, as well as access to the internet, whether they can even look up their symptoms, I could go on for hours.” (Provider 6, Emergency Medicine)*
Help-seeking	Access to care	*“I think financial sometimes is an issue. They know it’s gonna cost them a lot of money and they don’t have insurance, or they don’t have good insurance. That is sometimes an obstacle we see.” (Provider 2, Oncologist)*
Help-seeking; Diagnostic; Pre-treatment	Access to care	*“… language, that probably overlaps to some degree with socioeconomic dimensions; insurance, I think in this country is a big deal.” (Provider 3, Gastroenterologist)*
Diagnostic	Access to care	*“Payer issues, healthcare literacy, or communication barriers might stall or prevent accurate diagnosis when someone is lost to follow-up or does not show up.” (Provider 1, Internist)*
Diagnostic	Provider perception of patient risk	*“In the cases we have found and confirmed [ovarian cancer], [the patient’s symptom] is most commonly a feeling of abdominal fullness or feeling like they have a mass. And I feel like there has been one case of abnormal uterine bleeding which we didn’t think related back to the end malignancy finding but was [the symptom] [the patient] came in for.” (Provider 9, Gynecologist)*
Diagnostic	Features of complex diagnoses	*“I had gone to an instacare and they were like, ‘your lungs sound fine, I don’t know, maybe it’s walking pneumonia’ and they put me on antibiotics.” (Patient 8, Age 48, Stage III)*
Pre-treatment	Access to care; Social support	*“I asked [my gynecologist], “what do I do now?” She said, “well you call [Huntsman Cancer Institute] ...She looked at me and asked, “do you want me to do it?” I said yeah. She ended up making two calls to Dr. [Name removed] to get me in and the next week I was in Huntsman.” (Patient 14, Age 72, Stage III)*

#### Nonspecific symptoms

All ovarian cancer patients reported at least one symptom prior to their ovarian cancer diagnosis. First symptoms reported by patients included abdominal pain, urinary incontinence, back pain, urinary frequency, bloating, flatulence, cough, waxing and waning neck mass, weight gain, and tongue sores (Table 2). Most patients discussed a period of appraisal before help-seeking.

Patients and providers mentioned nonspecific symptoms as a barrier to earlier detection of ovarian cancer. Nonspecific symptoms reported by patients and providers included abdominal pain, general malaise, and discomfort.

*“A lot of the presenting symptoms for ovarian cancer are very vague, right? I mean, how many times have you had cramping? How many times have you felt bloated?” (Provider 2, Oncologist)*

#### Management and normalization of symptoms

Patient normalization of symptoms also delayed help-seeking and prolonged the appraisal interval. Patients who normalized their symptoms often attributed symptoms to indigestion, menopause, aging, a benign condition, or an existing medical condition.

*“With this particular cancer, you can discount all these warning signs … Oh it's just indigestion, or I've eaten wrong, or I've got gas and I shouldn't eat as many beans … you can go through and discount all those feelings.” (Patient 6, Age 57, Stage I)*

Many patients initially felt they could manage symptoms through lifestyle modifications and medications (e.g., eating more yogurt, taking tums, taking ibuprofen or other pain pills). Patients’ perceived capacity for self-management of symptoms and, in some cases, successful self-management of symptoms often prolonged the appraisal interval.

*“I already had pain pills from my pain doctors, so I would just take another pain pill and kinda self-medicate myself. I was going to school full time and working full time as well, and I didn’t want to stop and have to go to a doctor.” (Patient 7, Age 46, Stage I)*

#### Perception of underlying health

Patients’ evaluations of their own health also modified their appraisal of symptoms. Five of the 14 ovarian cancer patients described either a recent physical exam at their primary care provider’s office, a recent cancer screening (e.g., pap smear, colonoscopy, mammography), or both. For some of these patients, undergoing a physical exam or cancer screening accelerated their path to an ovarian cancer diagnosis, while, for others, normal findings from a recent cancer screening or preventative healthcare visit contributed to a personal perception of good health.

*“I had a complete physical in March: colonoscopy, and mammogram, and blood work. I was feeling absolutely great.” (Patient 10, Age 69, Stage IV)*

### Help-seeking

Themes identified across patient and provider interviews during the help-seeking interval included: worsening, extreme or abnormal symptoms, perceptions of underlying health, an avoidant coping strategy, persistence in seeking care, access to care, concerns about the patient-provider relationship, and social support (Tables 1 and 3).

#### Worsening, extreme, or abnormal symptoms

Abnormal or extreme symptoms, or worsening of existing symptoms, motivated nine of 14 individuals to seek medical care. Symptoms that prompted help-seeking included abdominal pain, difficulty breathing, post-menopausal vaginal bleeding, abnormal period, urinary frequency, bladder discomfort, incontinence, bloating, weight gain, dry heaving, cough, back pain, abdominal mass, neck mass, and lump in armpit (Table 2). Some symptoms (abdominal pain, dry heaving, and cough) were severe enough to prompt immediate care at an emergency department or urgent care, while others prompted visits to a primary care provider (PCP) or gastroenterologist.

Several patients described seeking medical attention because they knew, through either formal medical education or through being aware of their own bodies, that a symptom they were experiencing was abnormal and needed evaluation.

*“I worked in gynecology. I know post-menopausal bleeding needs to be worked up. So, I was like [my vaginal bleeding] is not normal.” (Patient 2, Age 53, Stage II)*

In contrast, other patients expressed frustration that they had never been taught about ovarian cancer symptoms or were slow to act when they sensed something might be wrong with their body.

*“I think I should have listened to my body, and I didn’t do that.” (Patient 12, Age 78, Stage IV)*

#### Avoidant coping strategy, and persistence in seeking care

Patients and providers noted that a patient’s inclination to seek medical care can be modified by their family history and fear of a serious medical diagnosis.

*“I really hadn’t been to a doctor for a very long time … if you go to a doctor, they’ll find something wrong, so I chose not to do anything.” (Patient 6, Age 57, Stage I)*

Providers also described patient persistence in seeking help as a facilitator of a timely ovarian cancer diagnosis.

*“… you've really got two different populations. [There is] the population of patients that pops out and wants to get seen and scanned right away, and then you have the other side of the coin where they're like ‘I don't want to know,’ [or] ‘I am afraid of what they're going to tell me’ so they don't report their symptoms, or they don't come in…” (Provider 5, Neurologist)*

#### Access to care and concerns about the patient-provider relationship

Access to care acted as a facilitator to help-seeking, while lack of access to care was a barrier. For five of the 14 patients, routine primary care appointments provided the opportunity to bring up abnormal symptoms.

*“I asked her about the lump because that's what you do when you go in for regular checkups. Just ask about all the little things that have been bothering you.” (Patient 9, Age 70, Stage IV)*

For other patients, even when symptoms prompted alarm, financial concerns, provider availability, patient work or school schedules, concern about judgement from providers, inability to find a provider with whom they felt comfortable, embarrassment about symptoms, fear of medical tests, and fear of a serious medical diagnosis were barriers to seeking help.

*“I didn’t want [my pain doctor] to think I was being a hypochondriac and trying to get more pain pills from them, so I never told them about [my new pain].” (Patient 7, Age 46 Stage I)'*

*“I really had not seen a primary care physician for a very long time. My issue was finding one that I would feel comfortable with. I did look up some, but a lot of them were not accepting patients, you know,* ‘*cause they're so busy.” (Patient 6, Age 57, Stage 1)*

Providers also mentioned that financial concerns and scheduling can be a barrier to patient help-seeking. In addition to factors mentioned by patients, providers named fear of social stigma, distrust in the medical system, dislike of doctors’ visits, language, and insurance as potential barriers to a timely cancer diagnosis.

#### Social support

Both patients and providers reported social support as a facilitator for help-seeking, including through encouragement to schedule and go to doctors’ appointments and help with transportation and childcare.

*“I said [to the trainer at the gym] ‘I just feel off. I feel tired. I don’t feel great. I don’t know. Something’s weird.’ And [the trainer] looked at me and she said ‘you need to go to the doctor,’ and I said, ‘I am not sick, I’m fine,’ and she said, ‘nope, you will not leave this building until you promise me that you’ll go to the doctor. Something is not right.’” (Patient 13, Age 29, Stage III).*

### Diagnostic

Themes identified across patient and provider interviews during the diagnostic interval included features of rapid and complex diagnoses, provider perception of patient risk, and access to care (Tables 1 and 3).

#### Features of rapid and complex diagnoses

The patient-reported diagnostic process typically began with either a trip to an emergency room or free-standing urgent care, or a visit to an established care provider (Table 2). When the diagnostic interval began in an urgent care or emergency room, same-day abdominal imaging was common, leading to a more rapid diagnosis.

Among patients who sought care from an established care provider, rapid diagnoses occurred when physicians were quick to order imaging. Informative imaging ranged from an x-ray to an ultrasound or computed tomography (CT) scan. Longer diagnostic intervals occurred when patients received evaluations and treatments for multiple incorrect diagnoses before ultimately receiving their ovarian cancer diagnosis (typically through imaging). For these patients, persistence in seeking help for reevaluation of symptoms acted as a facilitator to reduce their time-to-diagnosis of ovarian cancer since they often had multiple tests, office visits, and referrals to different specialty providers before receiving a workup for ovarian cancer. We did not observe longer diagnostic intervals for individuals presenting to primary care providers compared to specialists.

Patient-reported experiences of evaluation and treatment for differential diagnoses included treatment with antibiotics for a suspected infection related to abnormal white blood cell count, evaluation for pregnancy prompted by weight gain, and treatment with antibiotics for walking pneumonia related to a cough. One patient with urinary incontinence and gastrointestinal symptoms had a diagnostic interval that lasted more than 3 years, involving multiple self-referrals to gastroenterology and a naturopathic provider. This patient was worked up for irritable bowel syndrome (IBS) before palpating her own mass and requesting imaging.

*“I sought out a gastroenterologist … and [the gastroenterologist] just thought that I was having IBS … and that I needed fiber and probiotics and prebiotics.” (Patient 5, Age 55, Stage IV)*

Many providers described how ovarian cancer can be difficult to diagnose because the disease often presents with nonspecific symptoms that could have many underlying causes. Some providers noted that these nonspecific symptoms could lead a woman to seek care multiple times from the same provider or multiple times from a range of providers (e.g., primary care, pulmonology, gastroenterology) before receiving a diagnosis. Providers uniformly described imaging, either by ultrasound or CT scan, as critical to diagnosing ovarian cancer, though one provider noted that *“primary care doctors are trained to say ‘hey let’s triage this...’ and not just scan everybody all the time.” (Provider 2, Oncologist).* Providers noted patients’ persistence in seeking help or reevaluation acts as a facilitator to diagnosis.

*“I think a primary care doctor would eventually, if the person kept coming back with symptoms, eventually have to scan them and help make a diagnosis.” (Provider 2, Oncologist)*

#### Provider perception of patient risk

Most providers noted that, apart from a patient describing that she felt like she had a mass, symptoms alone may or may not increase their index of suspicion for ovarian cancer. However, symptoms that providers identified as most concerning for an ovarian cancer diagnosis included unexplained abdominal pain, weight loss, fevers, night sweats, abdominal distention, vaginal bleeding, patients feeling like they have a mass, and gastrointestinal abnormalities. In many cases, providers described that a combination of more than one of these symptoms would be most concerning. Contextual factors providers described as informative to the diagnostic process included: age, reproductive history, use of hormones, family history, and genetics. When asked if a patient’s help-seeking frequency increased or decreased concern for an ovarian cancer diagnosis, most providers mentioned that help-seeking behaviors have many origins, and their concern would depend on other factors, including if a patient had received any prior workup. Some providers also acknowledged that many patients do not like to come to the doctor, so when patients keep coming back that is worrisome.

*“My general philosophy in healthcare is that patients don’t like going to the doctor, so anytime I see a patient going to the doctor [I] have to wonder what we are missing … in general, if people show up on my doorstep, I always want to find out what's wrong with them, and even if I can't find it, I keep on digging.” (Provider 4, Gastroenterologist)*

#### Access to care

For some patients, social connections increased the accessibility of specialty providers, thereby accelerating the path to diagnosis. For others, limited access to specialty providers and financial concerns slowed their path to diagnosis.

*“I made an appointment [for the CA-125 test]... I thought okay if this is less than 200 dollars I will go ahead and do it. If it’s more than 200, it’s not worth it.” (Patient 14, Age 72, Stage III)*

Facilitators of a more rapid ovarian cancer diagnosis that were commonly referenced by providers included an established relationship with a primary care provider, access to specialists, and social support. Barriers to diagnosis that were commonly referenced by providers included insurance or financial barriers, distrust in the healthcare system, language barriers, and lack of accessibility of specialty providers. Multiple providers mentioned that primary care physicians are less familiar with ovarian cancer as a differential diagnosis, which can lengthen the diagnostic interval (though we did not observe this in the current study). Some providers also mentioned that, as specialists, it is common to give greater weight to differential diagnoses within one’s own specialty.

*“I would say that the biggest barrier is the lack of knowledge that most specialist providers have, and I would even say primary care doctors have, with female anatomy, pathology, et cetera … it's a little bit taboo, and because of that, it's not talked about as much and probably less discussed in medical circles.” (Provider 7, Cardiothoracic Surgeon)*

### Pre-treatment

Common themes identified across patients and providers during the pre-treatment interval were access to care and social support (Tables 1 and 3).

#### Access to care

For 13 of 14 patients the pre-treatment interval, defined from the time of ovarian cancer diagnosis to the start of treatment for ovarian cancer, was less than 2 weeks. Rapid access to treatment was facilitated by relationships between doctors’ offices, a strong social support network, hospital-based patient navigators, cancer center insurance and financial support programs, and travel support programs. However, one participant experienced an insurance-related treatment delay of over 6 weeks. Regardless of the rapid or delayed start of treatment, patients experienced anxiety about: (1) paying for treatment, and (2) the distance from home to their cancer treatment facility. Multiple participants mentioned needing to stay overnight to receive treatment and three participants reported traveling > 400 miles to receive care at Huntsman (presenting from Wyoming, Nevada, and New Mexico). Patients described feeling supported by cancer center programs that helped connect them with insurance and covered the costs of overnight stays.

Common barriers to care reported by providers during the pre-treatment interval included lack of trust in the health care system, language barriers, insurance barriers, and inability to pay for treatment. One provider described an experience during residency:

*“When I told [the patient] that [she had ovarian cancer], her response was ‘you just killed me’ because … she had no way to pay for further work up in care. And as far as I know … she never re-presented to our facility.” (Provider 6, Emergency Medicine)*

#### Social support

Both providers and patients mentioned social support as a facilitator in the pre-treatment interval. For patients, social support, especially from family, was essential to both their mental health and their access to care. Multiple patients expressed gratitude that a family member or friend had helped them with the paperwork or scheduling for their first oncology appointment. Family also helped with decision-making regarding where to go for care and supported the patient’s access to care by accompanying their loved one to appointments.

*“My daughters and husband called Huntsman and got me an appointment there, and I was so grateful.” (Patient 12, Age 78, Stage IV)*

## Discussion

This qualitative study focused on the patient path to ovarian cancer diagnosis as described by patients and providers in a US-based multi-payer healthcare system. In contrast to prior record-based studies, we were able to characterize time intervals that occur prior to the patient entering the healthcare system. These early time intervals (i.e., appraisal and help-seeking) contributed the most time to delays in diagnosis (Table 2), suggesting that interventions to shorten the appraisal and help-seeking intervals may be strategically important to decreasing the time-to-diagnosis for ovarian cancer in order to improve morbidity and mortality outcomes.

Interventions reported to shorten the appraisal interval for other cancer types have included increased utilization of population screening tools and symptom awareness interventions [10]. However, at this time, there are no effective population-level screening tests for ovarian cancer [6, 20, 31]. Given the lack of an effective population-level screening test for ovarian cancer, it is important to consider if symptom awareness could shorten the ovarian cancer appraisal interval.

Observations from our study suggest that ovarian cancer symptom awareness interventions may be well received by individuals at risk for ovarian cancer; patients, particularly those who waited a long time before seeking care, expressed disappointment that they had not received education about ovarian cancer symptoms. While patient education specific to ovarian cancer symptoms (e.g., bloating, abdominal pain, increased urinary frequency) could lead to more rapid ovarian cancer diagnoses for a small number of women [54], it is important to acknowledge that this benefit may be offset by added anxiety and distress among the larger population of women with ovarian cancer symptoms due to a benign condition. A study evaluating the predictive value of symptoms estimated that among women with ovarian cancer symptoms in the general population only 1 in 100 have ovarian cancer [39]. A less anxiety-provoking, but potentially effective, alternative to patient education on ovarian cancer symptoms could be patient education surrounding normal, age-related changes in health, to increase patient self-advocacy. Findings from our study and two UK-based qualitative studies indicate that education on these age-related changes could be useful for reducing symptom normalization, especially around the time of menopause [12, 28]. Positive self-perceptions of aging have been associated with higher likelihood of seeking primary care [25], and studies have observed associations between age-related stereotypes (e.g., attributing disease symptoms to the normal aging process) and increased all-cause mortality [41, 45]. Age stereotype interventions have been reported to improve the physical and mental function of aging individuals [24], and may inform symptom appraisal.

Targeted physician education on long-standing ovarian symptom indices (e.g., the Goff symptom index) has been suggested by other studies as a potential intervention to decrease time-to-diagnosis for ovarian cancer [19], but it is important to note that the Goff symptom index has relatively low sensitivity (65.5%) and specificity (84.7%) [13, 22] and has not been incorporated into clinical practice. In this study all providers were aware of at least one symptom included in the Goff index and the majority of patients (11/14) reported symptoms included in the Goff index [13], yet most providers were uncertain if symptoms alone would increase their index of suspicion for ovarian cancer over other differential diagnoses. Additionally, multiple providers stated that primary care providers’ limited consideration of ovarian cancer as a differential diagnosis could prolong the diagnostic interval. Interestingly, this perception was not consistent with results from a prior US-based quantitative study that found time-to-diagnosis was shorter for patients who initially presented to primary care physicians when compared to patients who initially presented to gastroenterologists or urologists [19]. While our sample size limited the ability to evaluate variation in time-to-diagnosis by provider specialty, we did not observe that time-to-diagnosis was consistently longer when a patient’s initial visit was with a primary care provider (Table 2).

Multiple providers mentioned that specialists give greater weight to differential diagnoses within their specialty, delaying referrals to specialists outside of their field. This was reflected in patient-reported pathways to diagnosis, including a patient who recounted an IBS workup with gastroenterology that lasted over 4 years. Some providers also mentioned that specialists outside of the gynecologic field were uncomfortable assessing gynecologic symptoms, pointing to the need to normalize conversations about pre- and post-menopausal gynecologic healthcare in all medical circles, starting with medical education.

Patient concerns about the patient-provider relationship acted as a barrier to initial help-seeking and help-seeking persistence. These concerns included fear of judgement from providers, embarrassment about one’s symptoms, and difficulty finding a provider with whom the patient felt comfortable. The patient-provider relationship is complex; however, compassion and communication training and reduced use of jargon by providers may improve the patient’s perception of the patient-provider relationship [36]. In our study, we noted that the language used to describe symptoms and experiences differed between patients and providers. Narratives of patient experiences can inform patient-provider communication interventions by providing references for terminology commonly used and understood by patients. Additionally, prior studies have reported that the way a provider communicates modifies the patient’s perception of risk [46]. In our study, patients noted that reassurance from a provider that a condition was benign led to normalization of symptoms and reduced help-seeking behavior. This finding is consistent with a prior observation of a positive association between greater gynecologic symptom concern and increased odds of help-seeking [49].

Narratives from both patients and providers suggested that the help-seeking, diagnostic and pre-treatment MPT intervals could be shortened with improved access to care. For 5/14 patient participants in our study, routine appointments with primary care providers facilitated a more rapid diagnosis by presenting an opportunity to bring up symptoms that the patient may not have discussed otherwise. Patient-perceived barriers to care included the timing, location and availability of appointments, financial barriers, and insurance issues. Providers also emphasized that for some patients access to care was a major barrier to timely diagnosis of ovarian cancer. While competing demands and provider availability were also reported as barriers to timely diagnosis in UK-based studies [12, 28], financial barriers and insurance issues were unique to our US-based study. Prior studies have suggested that offering after-hours care and increasing reliance on nurse practitioners may improve availability of care [44]. Policy-level interventions including the Affordable Care Act (ACA) and Medicaid expansion may also reduce the time to cancer diagnosis in the US multi-payer healthcare system, particularly for non-elderly patients and underserved populations [16, 29, 40]. These system-level interventions could impact the help-seeking, diagnostic, and pre-treatment MPT intervals by making it easier to schedule and pay for guideline-concordant care.

Our study provides insights into when and why delays in ovarian cancer diagnosis may occur. These insights allowed us to posit interventions that may decrease time-to-diagnosis, but, without rigorous evaluation of these interventions, we cannot know if they would result in stage shifts that consequently improve survival, or quality of life. For example, in the Australian Ovarian Cancer Study, a survey-based case-control study, there was no association between shorter time-to-diagnosis and disease stage or survival [32]. Similarly, when the UK implemented an urgent referral system to try to reduce diagnostic delays for cancer patients, urgent referrals for patients with a palpable pelvic mass or suspicious pelvic mass on ultrasound were associated with decreased time to seeing a physician, but there was no impact on stage at diagnosis or survival [33]. Even if reduced time-to-diagnosis does not improve overall survival, results from a study out of Denmark suggest that shorter time-to-diagnosis can be associated with greater quality of life and satisfaction after adjusting for age, stage, treatment status, and type of treatment received [38].

Strengths of our study included the qualitative nature of the study, which allowed us to characterize the appraisal and help-seeking intervals, and the inclusion of a range of provider perspectives, which allowed us to consider patient and provider interactions across different referral pathways. While the generalizability of our study was limited because we recruited patients from an academic tertiary care center, our patient population included individuals referred from Veteran’s Administration systems and both rural and urban community-based practices. Another possible limitation was the reliance on patient reports for calculating the time-to-diagnosis and time in each MPT interval. This may have introduced error in our estimates; however, relying on patients rather than medical record systems allowed us to consider medical encounters that occurred across multiple medical facilities and systems.

Interventions are urgently needed to shorten the pathway to diagnosis for ovarian cancer, in order to improve quality of life and potentially survival [38]. Our study expands on prior work by identifying patient and provider reported barriers and facilitators of timely ovarian cancer diagnosis in the US across the MPT intervals, including during the appraisal and help-seeking intervals. While prior quantitative studies indicated that reducing the time to an ovarian cancer diagnosis did not improve stage at diagnosis or ovarian cancer survival, these studies focused on the diagnostic and treatment intervals only [32, 33]. Our findings suggest that shortening the appraisal and help-seeking intervals may allow for a more dramatic reduction in time-to-diagnosis. Further studies are needed to understand if shortening the appraisal and help-seeking intervals can reduce time-to-diagnosis and improve survival and quality of life for ovarian cancer patients.

## Supplementary Information

**Additional file 1: Supplementary Information 1.** Patient semi-structured telephone interview questions.

**Additional file 2: Supplementary Information 2.** Provider semi-structured telephone interview questions.

## Data Availability

This study analyzed patient and provider transcripts. The original transcripts contain potentially identifiable information. All quotes analyzed in the qualitative analysis with extracted PHI information are available from the corresponding author on reasonable request.

## Abbreviations

US: United States
UK: United Kingdom
MPT: The Model of Pathways to Treatment
TCC: Total Cancer Care Study
EHR: Electronic health record system
FIGO: International Federation of Obstetrics and Gynecology
CT: Computed tomography
IBS: Irritable bowel syndrome
CA: Cancer Antigen
UKCTOCS: UK Collaborative Trial of Ovarian Cancer Screening
PLCO: The Prostate, Lung, Colorectal, and Ovarian
ACA: The Affordable Care Act
