# Supplementary material for: Pathways to ovarian cancer diagnosis: a qualitative study
Source: BMC Womens Health. 2022 Nov 4;22:430. doi: 10.1186/s12905-022-02016-1 (PMC9636716; doi:10.1186/s12905-022-02016-1)
Supplement: Supplementary file 1 — Additional file 1: Supplementary Information 1. Patient semi-structured telephone interview questions. [file 12905_2022_2016_MOESM1_ESM.docx]

**Supplementary Information 1:** Patient telephone introduction to the study and semi-structured interview questions.

| *Introduction to study (generalized to include all cancer patients):*  *We would like to mention a study you are eligible for. The study looks at healthcare utilization in <type of cancer> patients prior to their diagnosis including physician consults, medications, and diagnostic tests. It aims to identify patterns in healthcare usage in order to diagnose future <type of cancer> patients at an earlier stage. As part of the study we would ask you a few questions about your healthcare usage. Due to the large time period being asked about, we understand you won’t be able to answer all the questions, but we would be interested in hearing as much detail as you could provide. Would you be willing to participate in this study?* |
| --- |

**Patient Cancer History**

| 1. **BACKGROUND/TIMELINE** |
| --- |
| 1. What type of cancer were you diagnosed with? |
| 1. When were you diagnosed with <type of cancer>? __ __ / __ __ / __ __   MO. / DY. / YR. |
| **We are interested in learning about cancer patients’ experiences in getting to their diagnosis. We would like to hear your entire story including physicians you saw, medications you took, tests that were run, and any difficulties you faced in your journey.**   1. Could you tell me about your cancer story?    1. *Be sure to answer the questions of When, What, and Why for all of the following in the patient’s history: Doctors, Symptoms, Alternative healthcare providers, Medications, Tests, Reasons for having done everything (on patient and provider side)*       1. *Answers to these questions may be added into the additional subheading as we go through the interview.* |
| 1. What drove you to seek care initially? |
| 1. **SYMPTOMS** |
| 1. Tell me about the symptoms you experienced during the year prior to your diagnosis. What did you experience, when did you experience them, and how did you manage the symptoms (see a doctor, get medication, etc.)? |

| 1. **PHYSICIANS** |
| --- |
| 1. Tell me about the doctors you saw in the year prior to your diagnosis. When did you see them, how often did you see them, and why did you see them? |
| 1. What primary care physicians do you see, and how often do you see them? If none, write none. *(includes: family practice, internal medicine, OB/GYN, etc.)* |
| 1. Did you have a primary care physician throughout the year prior to your diagnosis? Why or why not? |
| 1. Did you ever go to an urgent care or free standing emergency room prior to your diagnosis? |
| 1. **ALTERNATIVE HEALTH PROVIDERS** |
| 1. Did you consult with any other healthcare providers in the year prior to your diagnosis (such as a chiropractor, physical therapist, dietician, massage therapist, or acupuncturist)? If so, who did you consult, when did you consult them, how often did you consult them, and why did you consult them? |

| 1. **MEDICATIONS** |
| --- |
| 1. Did you use any new medications in the year prior to your cancer diagnosis? If so, what did you use, when did you use it, how often did you use it, and what was it used for? |

| 1. **DIAGNOSTIC TESTS** |
| --- |
| 1. In the year prior to your diagnosis what, if any, tests do you remember having done on you? Why were they done, who requested them, when were they done, and what were the results/outcomes of those tests? |
| 1. **CARE QUALITY** |
| 1. Was there anything that happened during your experience you think could be improved for future patients? |
| 1. Was the cost of healthcare ever an obstacle to you receiving care? |

| 1. **RURAL ACCESS QUESTIONS**   *The following questions are intended for cancer patients living in rural areas and receiving care at Huntsman.* |
| --- |
| 1. How close is the nearest hospital to your house (in miles)? |
| 1. Is there a healthcare provider in your town? If so, how many providers and what type of providers are they? |
| 1. How far did you have to travel to get specialty help? (specialist examples include: gastroenterology, oncology, ENT, pulmonology, cardiology, etc.) |
| 1. Did you have to stay overnight to receive your cancer treatments? |
| - 1. Where did you stay if you had to stay overnight to receive cancer treatment? |
| 1. How do you afford your healthcare treatments and visits? (insurance, out of pocket, etc.) |
| 1. What difficulties did you encounter in receiving a diagnosis and getting treatment due to your rural location? |
