# Supplementary material for: Pathways to ovarian cancer diagnosis: a qualitative study
Source: BMC Womens Health. 2022 Nov 4;22:430. doi: 10.1186/s12905-022-02016-1 (PMC9636716; doi:10.1186/s12905-022-02016-1)
Supplement: Supplementary file 2 — Additional file 2: Supplementary Information 2. Provider semi-structured telephone interview questions. [file 12905_2022_2016_MOESM2_ESM.docx]

**Supplementary Information 2:** Provider telephone introduction to the study and semi-structured interview questions.

| **Introduction**: *Thanks for participating in our study. We’re interested in learning about patterns of care leading up to <type of cancer> diagnoses. Our goal today is to gain an understanding of your typical practices regarding patients who may have <type of cancer>.* |
| --- |
| **Section A: Patient Populations** |
| 1. About how many patients do you typically see each month? |
| 2. What types of patients or conditions do you see most frequently in your practice? |
| 3. In your practice, how many times would a typical adult patient come in over the span of 1 year? Is there a lot of variation by age, existing diagnoses, or other factors? |
| 4. About what percentage of your patients are female? |

***Sections B1-D1 and B2-D2 may be completed consecutively or concurrently, whichever is the preference of the provider.***

| **Section B1: Presentation of <<type of>> Cancer** |
| --- |
| 1. Using knowledge from your personal and professional experiences, how would you describe the most common start to finish pathway to <<type of>> cancer diagnosis? |
| 2. Have you ever to your knowledge treated a patient who was later diagnosed with <<type of>> cancer?   1. If yes, what was your role in that patient’s cancer diagnosis? 2. Did you continue to see that patient after his or her diagnosis? 3. Were you involved in his or her cancer treatment? 4. Did you continue to see him or her in between or after the conclusion of their cancer treatment? |
| 3. What presenting symptoms do you associate with <<type of>> cancer? |
| 4. Which of these symptoms are most alarming to you or most indicative of a potential <<type of>> cancer? |
| 5. When you see a patient presenting with these symptoms, what do you do next? |
| **Section C1: Patient Behaviors for <<type of>> Cancer** |
| 1. Does an increase in visit frequency raise or lower your index of suspicion for <<type of>> cancer? |
| 2. Are there any patient behaviors that raise or lower your index of suspicion for <<type of>> cancer? |
| **Section D1: Other for <<type of>> Cancer** |
| 1. What other factors do you think might influence a patient’s pathway to a <<type of>> cancer diagnosis? |
| 2. Have you noticed any obstacles in a typical patient’s pathway to a << type of>> cancer diagnosis? |

| **Section B2: Presentation of <<type of>> Cancer** |
| --- |
| 1. Using knowledge from your personal and professional experiences, how would you describe the most common start to finish pathway to <<type of>> cancer diagnosis? |
| 2. Have you ever to your knowledge treated a patient who was later diagnosed with <<type of>> cancer?   1. If yes, what was your role in that patient’s cancer diagnosis? 2. Did you continue to see that patient after his or her diagnosis? 3. Were you involved in his or her cancer treatment? 4. Did you continue to see him or her in between or after the conclusion of their cancer treatment? |
| 3. What presenting symptoms do you associate with <<type of>> cancer? |
| 4. Which of these symptoms are most alarming to you or most indicative of a potential <<type of>> cancer? |
| 5. When you see a patient presenting with these symptoms, what do you do next? |
| **Section C2: Patient Behaviors for <<type of>> Cancer** |
| 1. Does an increase in visit frequency raise or lower your index of suspicion for <<type of>> cancer? |
| 2. Are there any patient behaviors that raise or lower your index of suspicion for <<type of>> cancer? |
| **Section D2: Other for <<type of>> Cancer** |
| 1. What other factors do you think might influence a patient’s pathway to a <<type of>> cancer diagnosis? |
| 2. Have you noticed any obstacles in a typical patient’s pathway to a << type of>> cancer diagnosis? |
